# Supplementary material for: Are carbon emissions trading and green financial instruments synergistic? -Comprehensive quantitative research based on content analysis
Source: PLoS One. 2024 Mar 7;19(3):e0298601. doi: 10.1371/journal.pone.0298601 (PMC10919601; doi:10.1371/journal.pone.0298601)
Supplement: S1 Data — (ZIP) [file pone.0298601.s001.zip › Data/地级市Z_F工作报告.zip!/║■─╧/╔█╤⌠╩╨/└┤╫╘╣1⁄2╓┌║┼í╛┬φ┐╦╩2╛▌═°í┐_2015.docx]

各位代表：

现在，我代表市人民政府向大会作工作报告，请予审查，并请各位政协委员和其他列席人员提出意见。

一、2014年工作回顾

过去的一年，在省委、省政府的坚强领导下，我们按照中共邵阳市委的决策部署，在市人大、市政协的监督支持下，解放思想，攻坚克难，努力适应经济发展新常态，较好完成了市十五届人大二次会议确定的目标任务。

1、经济稳步快速发展。努力克服宏观形势偏紧、经济下行压力增大等多重困难，经济发展稳中快进。主要经济指标全部实现两位数增长，增幅全部高于全省平均水平。实现地区生产总值1261.6亿元，增长10.8%，高于上年0.6个百分点，高于全省1.3个百分点，排全省第2位、中西部市州第1位。财政收入达到117亿元，增长12.2%，高于全省2.7个百分点，排全省第5位。财政支出342.5亿元，比上年增加35.7亿元，增长11.6%。全面小康建设加快推进，39项指标已有7项达到目标值，全市总体实现程度78%，比上年提高5.2个百分点，我市获全省经济发展奖。万元生产总值能耗下降7.5%。隆回、洞口列为全省第二轮特色县域经济农副产品加工重点县。省委、省政府从全省战略布局出发，专门以五部门联合行文《关于加快邵阳湘商产业园区建设的若干意见》，给予十条优惠政策。这是全市人民翘首盼望的一件大事，具有历史突破和长远发展的重大意义。

2、产业发展提速增效。产业结构持续优化，三次产业比由上年的22.5:38.9:38.6调整为21.8:38.2:40。工业经济规模扩大。规模工业总产值1707亿元，增加值达491亿元，增长11.6%，高于全省2个百分点，排全省第4位、中西部市州第2位。产值过百亿产业4个，其中轻工产业过400亿，建材、食品产业均过200亿，机械产业180亿。“四百工程”超额完成任务，新开工项目137个，竣工投产项目135个，引进项目141个，新增规模企业176家、居全省第3位。新增小微企业3813户，增长87%。工业园区实现规模工业增加值313亿元，占全市规模工业增加值的63.8%，其中宝工区实现规模工业增加值45亿元，增长39%。农业现代化水平提升。农业总产值实现“十一连增”，达到404亿元，增长4.8%；农民人均可支配收入实现“十一连增”，达到7786元，增长12.3%，高于全省0.9个百分点，排全省第1位。粮食播种面积稳定在900万亩左右，总产达321万吨，增产9.2万吨。经济作物播种面积246万亩，新增1万亩，总产值18.5亿元。收购烟叶22.9万担。出栏生猪985万头、肉牛60万头、山羊84万只、家禽5641万羽，分别增长3%、2%、3%和0.5%；发展水产品10.6万吨，增长3%。新增国家五星级农庄1家、农业产业化企业20家、农产品基地9.8万亩、农民专业合作社60家。新建新农村重点村22个、畜禽标准化养殖场80个。推广农机具4.5万台(套)，综合机械化水平达58%，提高4个百分点。现代服务业加速发展。新增限额以上企业67家，实现社会消费品零售总额488亿元，增长13%，高于全省0.2个百分点，排全省第1位。电子商务、互联网、金融等新兴业态加快发展，洞口雪峰蜜桔在渤海交易所整体挂牌上市，中国卤菜交易网、老百姓易购网、邵东购、宝庆佬农产品网上商城等一批电商品牌进入市场，邮政快递、电子商务以50%的速度增长，移动4G网络成功实现商用。编制完成旅游发展总体规划和西部生态圈文化旅游规划。崀山创5A景区通过国家景观质量评审，新宁成为湖南首个全国旅游标准化示范县。成功举办2014中国湖南国际旅游节、南山六月六山歌节等有影响、有实效的旅游促销活动。接待游客2257万人次，增长18.8%；实现旅游综合收入167.4亿元，增长19.4%。新增3家商业银行、2家证券营业部、2家保险机构、4家小额贷款公司、9个融资性担保机构，农信社成功改制，邵阳农商银行春节前挂牌，6家企业在新三板等多层次资本市场挂牌。新增各类融资总额328.1亿元，增长48.6%。存款余额达到1736亿元，贷款余额达到752亿元，存贷比达到43.3%，比上年提高0.6个百分点。竣工商品房209万平方米、增长14.2%，商品房销售面积293万平方米、增长27.4%。房地产对税收贡献率达33.7%，比上年提高3.8个百分点。

3、项目建设可圈可点。完成固定资产投资1268亿元，增长23.1%，高于全省3.7个百分点，增速在全省排名实现第7、第4、第2的三年三大跨越。产业投资占固定资产投资的比重达39.5%，提高2.1个百分点。实施337个重点项目，实际完成投资495亿元，比上年增加90亿元，其中竣工投产项目72个，为历年之最，我市重点项目建设工作获省先进。产业项目方面：恒天九五完成厂房建设，湘窖酒业建好品酒车间和地下管网，李文食品进行生产线设备安装调试，武冈百威啤酒生产基地完成场地平整，三一湖汽混凝土搅拌车基地抓紧配套设施施工，云峰水泥与海螺水泥成功合作。九兴鞋业市生产基地正式投产，总部基地完成场地平整，新宁基地投产，邵阳县基地正在招工即将投产。平板超薄超强玻璃、统一等项目已完成征地拆迁和场地平整。交通项目方面：沪昆高铁邵阳段通车运营，高铁邵阳北站站房及广场投入使用，标志着我市正式跨入高铁时代。娄邵铁路扩改进入路基建设，怀邵衡铁路全线征拆和开工，兴永郴铁路可望进入“十三五”规划，常桂海铁路有望进入中长期铁路规划。邵坪高速超额完成年度投资计划，武靖高速基本完成征地拆迁，控制性工程已提前开工。新改建干线公路35.4公里，新开工231.2公里，完成农村通畅工程642.4公里，改造危桥79座，渡改桥1座。武冈机场获国家发改委正式批复，场内外已全面开工建设，省机场管理公司提前进驻。城建项目方面：城市总体规划和东部城镇群城镇体系规划正式呈报省政府，市区控规覆盖率达100%。实施中心城区重点路网及配套项目136个，其中续建75个、新建61个，完成投资66.3亿元。项目多、投入大，建设体量为历史之最。中心城区120平方公里土地利用规划正式获批。全市新增建设用地19450亩，其中市本级7420亩。市城建投年内融资44.45亿元。东互通世纪大道、南互通敞口工程、邵石南路、大祥立交桥等建成通车，“二桥二公园二隧道”、学院路二期、邵坪L2连接线等进展较快，“最美十条街”改造全面铺开，西湖北路完成提质改造。宝庆新城、桃花新城、江北新城路网工程加速推进。人防1213工程主体完工。农田水利项目方面：实施娄邵盆地高标准基本农田建设项目27万亩，完成投资6亿元，新增耕地1469.2公顷。农业综合开发高标准农田示范建设项目进展顺利。水利建设五年行动计划强力推进，治理中型水库3座，整治重点小二型病险水库91座。崀山水库列为国务院加快建设的172个重大水利工程之一，已编制完成项目建议书。木瓜山水库改扩建工程完成测量、踏勘等工作，正在编制可行性研究报告。能源项目方面：能源建设全省领先。风电项目已建成6个、在建11个，共85.6万千瓦，总投资80亿元，超过全省的三分之一，建成和在建风电项目总量居全省第一。农村电网管理体制改革工作在全省率先完成，已有97个自供区593个村完成改革任务。农网改造和电网建设完成投资7.7亿元。社会项目方面：市一中搬迁、邵阳幼专建设、邵阳学院与邵阳医专合并工作扎实推进。市文化艺术中心“五馆二中心”完成主体工程，大剧院主体工程即将竣工。市体育中心主体育馆即将封顶，其他场馆建设已进入征地拆迁和场地平整。市一人民医院儿科综合楼、市精神病院门诊及住院综合楼投入使用，市中心医院东院门诊楼完成地下建筑工程，市二人民医院整体北迁项目已进入招商阶段。

4、改革开放全面深化。9大类56项改革全面启动，实现了新一轮改革的快速反应、平稳起跑。国企改革三年攻坚计划提前完成，市本级完成国有企业改制66户，筹措资金35亿元，安置职工4.5万人，实现了完成任务多、筹措资金多、化解矛盾多和总体效果好的“三多一好”，经验在全省推介。在全省率先平稳关闭落后小煤矿65家，被誉为“邵阳模式”，经验在全国推介。市县政府机构改革方案获省批复。市本级行政许可项目由301项精简到121项，非行政许可项目由150项精简到37项。注册资本登记制度和工商登记前置审批制度改革扎实推进，新登记企业5443户、增长47.1%，新增注册资本227.4亿元、增长62%。“三榜公示、两级审核、经费包干”的阳光征拆模式全面推行。完成农村土地承包经营权确权登记颁证试点，流转农村土地176万亩。大圳灌区改革完成。隆回国有林场改革经验在全国推介。实际利用外资1.7亿美元，增长22%，排全省第3位。实际利用市外境内资金737.3亿元，增长25.1%，排全省第3位。成功举办2014邵商大会，签约项目40个，合同金额113.4亿元。新增进出口实绩企业28家，有37家资质外贸企业实现“零突破”，新增境外投资企业25家。实现进出口总额8.4亿美元，增长42.5%，分别高出全国全省40和19.3个百分点。

5、创建工作成效显著。中心城市成功创建省级文明城市，市民文明意识不断增强，社会主义核心价值观深入人心，我市在全省文明指数测评中综合得分90分，名列评选城市前茅。成功创建省级园林城市，市区绿地率达到31.46%，绿化覆盖率达到35.12%，人均公园绿地达8平方米。成功创建省级双拥模范城，修缮涉军重点文物单位3处，开办国防教育课3000余次，建立军民共建点185个，双拥结对单位40对。创建国家卫生城市取得阶段性成果，新建公厕28座，提质改造778座，新建垃圾中转站30座，提质改造34座，改造升级农贸市场42家，已竣工使用28家。新增和更新公交站牌150座，候车亭40座，枫林公交停保场竣工投用。桂花渡水厂取水泵房改造主体工程竣工，江北污水处理厂试运行。拆除违法建筑36.7万平方米，违法建设不断减少。全面推行城市“牛皮癣”清理、干道清扫保洁、资江邵水垃圾打捞市场化运作，市容秩序、环境卫生、渣土运输、户外广告等专项整治效果明显。

6、民生事业取得新进步。公共财政用于民生方面的支出大幅增加，占一般预算支出七成以上。15项29个为民办实事项目全面完成，其中11项超额完成任务。城乡居民人均可支配收入达到11955元，增长10.7%，高于全省0.6个百分点，排全省第1位。居民消费价格涨幅控制在2%以内。就业和社会保障进一步加强。新增农村劳动力转移6.2万人、城镇就业5.1万人，零就业家庭就业援助达100%，失业率控制在4.3%以内。征收各项社保基金40.7亿元，增长20.5%，创历史新高，社保惠民一卡通在邵阳县首发。保障城乡低保对象693.9万人次。新改扩建敬老院14所。改造城市棚户区23282户、国有工矿棚户区1562户，新增公租房114万平方米，其中农村学校教职工和乡镇卫生院医务人员周转房2248套。完成农村危房改造16164户。归集住房公积金16.2亿元、增长36%，发放个人贷款12.7亿元、增长21%，实现增值收益1.1亿元，其中用于保障房建设6000万元。建成农村饮水安全项目280个，解决65万人饮水不安全问题。为近5万户残疾人家庭发放了机动轮车燃油补贴。片区开发及扶贫攻坚取得新成效。武陵山片区开发和湘西开发政策较好落实，完成投资779亿元。实施精准扶贫，完成115.5万贫困人口识别登记工作。派驻520个工作组进村扶贫，国家商务部进驻城步定点扶贫。全年减少贫困人口18万人。社会事业全面进步。规模以上工业企业研发经费达到13亿元，增长32.4%。获省科学技术奖6项，应用类科技成果转化率达80.5%。实现专利授权968件，增长20.6%。建成院士专家工作站4个。改扩建农村校舍60万平方米，建成农村公办幼儿园29所、义务教育合格学校155所，为农村寄宿制学校添置学生床位53190个，薄弱学校基本配齐标准桌椅。启动化解城区大班额三年行动计划，改扩建市区学校16所。“欢乐潇湘”群众文化活动节目在全省汇演中获得6个奖项。送戏下乡演出583场，放映农村公益电影6.6万场次，为5542个农家书屋配送图书240万册。洞口“棕包脑”、新邵孙氏正骨术和隆回滩头手工抄纸技艺被列为第四批国家级非物质文化遗产保护名录。全国第一次可移动文物普查基本完成。隆回获评全国文化先进县。绥宁侗寨申遗工作进展顺利。新增广播电视用户54623户。建成体育健身工程215套、室外健身路径工程22个。邵阳健儿在省以上竞赛中获金牌40块，其中国家级金牌2块。改扩建精神卫生服务机构1家，市中医院、中西结合医院、医专附属医院成功晋升三级医院。基本药物制度实施比率达到96.6%。新农合参合率达到98.3%。“单独两孩”生育政策得到落实，4000多名单独夫妇领取“两孩生育证”。为5.7万城镇独生子女父母发放奖励金5500万元。人口自然增长率控制在6.6‰以内，常住人口符合政策生育率81.2%，继续保持全省先进行列。社会大局安全稳定。扎实开展安全生产大检查和“六打六治”专项行动，没有发生重特大事故。沪昆高速“7·19”事故应急救援和善后处置的经验得到中央和省委、省政府肯定。认真落实领导接访、下访和包案责任制，化解信访积案326件。建成市（村）民法制学校3452所，经验在全国推介。218个社区警务场地问题得到解决，专职社区民警和辅警全部配齐。深入开展严打整治和综合治理，没有发生恶性刑事案件，没有发生大的群体性事件，命案数量大幅下降，命案破案率达98%；中心城区限摩限电成果得到巩固，市区抢劫、抢夺案件基本消除，群众安全感和满意率大大提升。我市综治民调居全省第八，创历史最好成绩。市公安局治安支队被评为“全国公安机关爱民模范”。城步苗族自治县成功创建全国民族团结进步模范县。

7、生态文明水平持续提高。“三边三区三年”绿色行动深入推进，植树造林39.6万亩。巩固退耕还林成果工程完成投资2.1亿元。森林覆盖率达到60.6%，比上年提高2.1个百分点。绥宁、武冈、新宁、邵东、邵阳县纳入全国第二批水土保持监督管理能力建设县。紫薇集团投资、设计和建造的“湖南园”荣获青岛世界园艺博览会室外展园竞赛特等奖。城步南山草原荣获“中国美丽田园”称号。新邵筱溪和洞口平溪江被评为国家湿地公园。实施地质灾害治理项目7个。取缔非法企业71家，停产整治违法排污企业50家。龙须塘化工区综合整治、市污泥集中处置、城区“煤改气”工作顺利推进。新建户用沼气池2400户，养殖小区和联户沼气工程255处。农村环境卫生综合整治强力推进，农村卫生状况大为改观。全市整体纳入全国生态文明示范工程试点。绥宁列入国家重点生态功能区转移支付补偿范围。隆回县虎形山获国家级生态乡镇称号。

8、政府执行力和公信力不断提升。扎实开展党的群众路线教育实践活动，聚焦“四风”，对作风之弊、行为之垢进行了大排查、大检修、大扫除，干部作风明显好转，行政效能切实提高。自觉接受市人大及其常委会的法律监督、工作监督，自觉接受市政协民主监督，与市政协民主协商，认真听取各民主党派、工商联、无党派人士和社会各界的意见和建议。对重大建议和提案及时形成政府决策并努力落实，办理人大代表建议176件、政协提案420件，办复率均为100%。扎实推进依法行政，建立完善市政府常务会议学法制度，举行重大行政决策听证会52次。政务服务和政务公开深入推进，公开了37个政府组成部门的财政预算和“三公”经费预算信息。审计项目277个，查处各类违规金额10亿元，管理不规范资金49亿元。加强政府廉政建设，查处了一批违法违纪案件。

第三次全国经济普查基本完成，移民、老龄、市志、档案、贸促、供销、盐业、石油、烟草、民族宗教、外事侨务、农民减负、气象水文、防震减灾、无线电管理和山林纠纷调处等工作都取得新的成绩。加强与工会、妇联、残联、文联、侨联、科协、共青团、社科联、红十字会等群众团体的联系。全面加强国防动员、民兵预备役和国家安全、人民防空工作，驻邵解放军、武警、消防部队和民兵预备役人员在抢险救灾、维稳处突和支持地方建设等方面做出了重要贡献。

各位代表，过去的一年，我们群策群力，风雨同舟，成绩来之不易。在此，我代表市人民政府，向全市人民，向各位人大代表、政协委员，向各民主党派、工商联、无党派人士和人民团体、离退休老同志，向驻邵人民解放军、武警官兵和政法干警，向所有关心、支持邵阳改革发展的各界人士，表示衷心感谢!

我们也清醒认识到，我市发展不快不足不优、总体实力偏弱的现状没有根本转变；部分指标没有达到预期；产业基础相对薄弱，综合生产能力和现代化水平有待提高；深化改革任务十分艰巨，改革的主动性、创造性、积极性有待增强；社会就业难度较大；安全生产形势仍然严峻；政府职能转变有待进一步深入，工作作风有待进一步改进，发展环境有待进一步优化。对此，我们一定采取更加有力的措施，切实加以解决。

二、2015年的形势、目标和主要工作

2015年是适应经济发展新常态的调整年，也是“十二五”和“十三五”规划的衔接年。一方面，我们面临更多困难。全球经济复苏疲弱态势难以明显改变。我国经济呈现增长速度换挡期、结构调整阵痛期、前期刺激政策消化期“三期叠加”的阶段性特征，经济下行压力会更大。经济发展新常态下，我市区位优势仍然不明显，靠投资拉动的压力增大，有效需求不足、资金土地要素偏紧、一系列关乎民生的路电水等基础设施任务重和体制机制老大难矛盾多的问题，都将对我市加快发展产生直接影响。另一方面，我们更面临难得历史机遇。国家大力实施“三个支撑带”战略，我省“一带一部”区位优势更加凸显，正在努力培育新的增长点、增长极、增长带。新常态带来的新机遇，将为我市带来更多投资机会、更多项目布局、更大市场空间。我市已实现县县通高速、进入高铁新时代、武冈机场明年正式通航，完全具备融入长三角、珠三角、成渝经济区、大西南地区的时空条件，将带来大量的人流、物流、信息流、资金流和项目流。省委、省政府作出在邵阳建设湘商产业园的重大决策，为我们带来了千载难逢的发展机遇。随着怀邵衡铁路、武靖高速、水利建设和棚户区改造五年行动计划等重大工程快速推进，既为经济发展注入强劲动力，也将不断提升民生的质量和水平。我们只要保持定力、精准发力，超前认识新常态，提前适应新常态，善于在新常态中捕捉新机遇，就能破除路径依赖、思维定势和工作惯性，就能在新常态下把握新一轮发展改革的主动权。

2015年政府工作的指导思想是：全面贯彻落实党的十八大、十八届三中、四中全会以及中央、省委经济工作会议精神，以全面建成小康社会和“八个建成”为总揽，始终坚持稳中快进总基调，紧紧抓住湘商产业园建设重大机遇，着力加快发展，壮大经济实力；着力深化改革，增强发展的内生动力和市场活力；着力保障和改善民生，累积人民福祉；着力推动绿色发展，提高生态文明水平；着力安全发展，提高人民群众生命的安全度，努力实现新常态下更有速度、更有质量、更有安全的发展。

经济社会发展预期目标是：地区生产总值增长10%以上，规模工业增加值增长12%以上，固定资产投资增长24%，社会消费品零售总额增长13%，财政收入增长12%以上，利用外资增长20%以上，利用内资增长25%，进出口总额增长25%，城乡居民收入分别增长10%、12%，城镇化率达到41%，人口自然增长率控制在7.8‰以内，居民消费价格涨幅控制在3%左右，万元生产总值能耗下降4%。

围绕上述指导思想和目标要求，市政府将始终坚持“五个不动摇”，即加快发展不动摇、突出项目建设不动摇、做大做强工业不动摇、深化改革不动摇、优化发展环境不动摇。以前瞻思维和务实态度，科学编制“十三五”规划，争取更多政策和项目上升到省级和国家级层面。全年实施重点项目318个，总投资3600亿元，年度投资600亿元，其中产业项目投资占投资总量的比重不低于40%，工业项目投资占产业项目投资总量的比重不低于60%。

主要抓好十个方面的工作：

1、把加快湘商产业园建设作为工作的重中之重

全力以赴推进湘商产业园建设。用好用活十大优惠政策，大胆先行先试，筑好巢引好凤。科学确定园区面积和产业定位，加快扩区调规进程。重点推进标准化厂房建设。进一步完善招商引资的考核机制，大力招商入园，高水平承办好第七届湘商大会。继续实施工业园区经济倍增工程。强力推进邵阳经开区（含宝工区）申报国家级经开区。

继续实施“四百工程”。引进、开工、投产、新增规模企业均在100家以上。更加注重质量，投资过亿元项目占开工、投产、引进项目总量的30%以上，高新技术产业和战略性新兴产业企业占入规企业的30%以上。完善部门单位联点帮扶企业机制，力争已有规模企业少停产或不停产。

推动传统产业和新型产业创新驱动发展。实施提升传统产业和培育新兴产业双轮创新驱动。支持机械、轻工、食品、建材等传统优势产业改造升级和兼并重组，加快创新发展，四大产业总产值过千亿元。促进新能源、新材料、电子信息等新兴产业健康发展，积极培育移动互联网、智能制造等新产业、新业态。发挥企业科技创新主体作用，引导和鼓励社会资本投入科技研发，切实提高科研经费占GDP比重，全社会科技投入增长20%以上。新增一批院士专家工作站和省市重点实验室、工程技术研究中心。实现专利申请1500件以上、授权800件以上。加快科技成果转化，新增高新技术企业6家，高新技术产业产值达到580亿元以上。

2、高度重视三农工作，促进农业持续增长

大力发展现代农业。推进三个“百千万”工程，实施现代农业科技示范园、百万亩特色产业发展、农村环境卫生整治等五年行动计划。确保粮食种植910万亩、总产340万吨。完善农业标准化体系，加快农业产业化。支持建设一批畜禽生产规模化、标准化健康养殖示范场。以粮油“百亿产业”工程为抓手，扶持茶油产业发展壮大，大力推进粮油深加工和副产品循环利用。启动小江湖粮库异地扩建工程和粮油农产品批发市场建设。

不断改善农业生产和服务条件。推进水稻生产全程机械化，农业生产综合机械化水平达到59%以上。继续实施娄邵盆地和农业综合开发高标准农田建设工程，建成高标准基本农田21万亩。做好土地经营权确权登记颁证工作，引导土地经营权有序流转。发展综合服务社，打造“网上供销社”。推进气象为农服务示范点、农村防雷示范工程、水文站建设，提高抵御自然灾害的能力和水平。组建市农业科学研究院。

突出重点抓好新农村建设。坚持规划先行，做好村庄布局规划编制工作。建好省级12个为民办实事新农村示范村和市级第四轮22个新农村重点示范村。大力发展村级集体经济，减少集体经济空白村500个。逐步增加乡镇（街道）、村（社区）的运转经费，建立工作经费正常增长机制。继续推进农村环境卫生综合整治，加强农村面源污染防治，开展畜禽养殖污染专项整治，建成市级标准卫生村1000个。建好户用沼气池2400户、养殖小区和联户沼气工程365处、大中型沼气工程9处。

3、增强第三产业活力，形成新的更多消费热点

加快发展商贸流通业。加快建设以湘西南国际商贸物流城为代表的现代商贸物流园区。推进县乡流通再造工程，完善农产品现代流通体系。加快步步高、友谊阿波罗、红星美凯龙等大型购物商场建设。鼓励各类市场主体开展网络商品交易活动，支持与网络交易配套的邮政、快递、支付、仓储等行业发展，新增网络市场主体1万户。新增限额以上企业100家以上。完善重点商标品牌梯次培育制度。推进外资、外贸、外经联动和内外贸融合发展。加快建设海关和检验检疫业务中心。

推进旅游产业快速发展。重点抓好崀山、南山、黄桑、云山、花瑶、溪、白水洞、蔡锷故居、紫薇博览园、湘商文化旅游产业园10大景区的规划和建设。加快崀山创5A景区步伐。按照“深耕省内、炒热武广、进军沪昆、打通港澳台、触及韩日”的思路，加强旅游产品营销，吸引更多游客来邵旅游，接待游客2700万人次以上，实现旅游综合收入180亿元以上。

积极推进金融业发展。健全多层次资本市场体系，帮助符合条件的企业上市和发行企业债券。积极引进金融机构到我市设立分支机构，新增商业银行2家以上。完成邵阳农商银行组建工作，新开业2家县级农商银行，新建村镇银行2家。支持成立融资性担保公司，完善融资担保体系。扶持小额贷款公司发展。抓好银企合作。存贷比达到44%以上。

4、积极稳妥推进新型城镇化

推动东部城镇群和西部生态圈建设。加快建设学院路二期，确保邵塘一级公路、邵坪高速L2连接线竣工通车。启动东部城镇群快速环线前期工作。高起点编制西部生态圈规划，高标准推进旅游快速通道和旅游项目建设。

加快中心城区扩容步伐，稳步实施提质工程。按照省域次中心城市、湘西及湘西南中心城市和“双百”规模山水园林城市定位，完善市区综合交通规划，抓好供水、燃气、环卫设施等专项规划的修改或编制工作。按照加快扩容、量力提质的工作总思路，突出路网拓城，启动三环快速路前期工作。开工建设白马大道、蔡锷大道。拉通邵水东路和邵水西路南延伸段、桂花桥、雪峰桥北段道路以及魏源西路。逐步完善宝庆新城、桃花新城、江北新城和宝工区路网。加快整体推进“最美十条街”速度。改造东大路、邵石北路、三眼井路、双清路，加快电机路东段、资江一桥南匝道、建设北路隧道、新华南路隧道等瓶颈路畅通工程建设。抓紧雪峰、桂花“二桥”与东塔公园、北塔公园、西苑公园、龙山公园、蔡锷纪念园“五园”建设。建成1213人防工程（爱莲广场），启动人防疏散基地建设。深化公交优先，提高公共交通出行分担率。进一步规范出租车行业管理。抓好城区供水管网新造项目和白马加压站建设，改造水质检测中心。完成洋溪桥污水处理厂扩改，建成市污泥集中处置中心。启动垃圾焚烧发电和餐厨垃圾处理项目。开工建设城区次高压储气环网项目，推进燃气管网配套工程和汽车加气站建设。

提高城市建设要素保障能力。全面完成中心城区和各县城区土地利用总体规划修编，挖掘存量建设用地潜力，尽力保障新增建设用地。完善征地拆迁安置改革细则，落实失地农民保障政策。推进市城投管理体制改革和理顺北塔城投体制，规范投融资行为。继续保持控违拆违高压态势，力争违法建设零增长，拆除存量违法建筑30万平方米以上。

全力创建国家卫生城市。在通过省暗访评估基础上，完成农贸市场、公厕、垃圾中转站等基础设施建设。强力推进空中缆线、城市“牛皮癣”、户外广告、违规饲养家禽家畜、市政道路、渣土“六大专项整治”。建成数字化城管系统，提高智能化管理水平。整治背街小巷、城中村和城乡结合部的环境卫生。进一步健全市容环境卫生常态管理机制，确保通过国家暗访及技术评估。将创建工作延伸至县城，促使各县城功能和品位有一个大的改观。

5、推动交通、水利、能源、通讯“四网”升级，夯实发展基础

加快构建立体交通体系。研究编制十三五交通建设规划，夯实区域性交通枢纽地位。确保娄邵铁路、邵坪和安邵高速公路竣工通车。加快怀邵衡铁路、武靖高速公路建设。争取邵永铁路扩改、新白高速公路开工。推进呼北高速新化至武冈、城步至龙胜高速前期工作。把干线公路和农村通畅公路建设摆在更重要的位置，改造干线公路3条、86公里，续建10条、307公里，开工8条、158公里。建成农村公路303公里。加快武冈机场建设，推进邵东军民合用机场前期工作。加快邵阳港、资江干流航道整治。建成邵阳高铁北站综合客运枢纽，启动邵阳南站综合客运枢纽、邵东汽车东站、新宁汽车北站、城步汽车南站建设。

深入推进水利建设五年行动计划。完成103座小型病险水库、3座大型病险水闸的除险加固，做好7座大中型病险水库和18座大中型水闸前期工作。加快东风水库建设，争取向阳坝水库、柳山水库立项。继续强力推进崀山水库、木瓜山水库前期工作。抓好“十二五”中小河流治理扫尾工作。加快“五小”水利工程建设，畅通“最后一公里”。加快市城区、县城、沿河乡镇城市防洪堤建设和邵水河、红旗河治理。彻底解决白云水库移民遗留问题。用超常规手段解决农民饮水不安全问题。

积极发展新能源。建成龙山、蒸乡、寨子背、大云山、牛排山等风电场，开工建设金紫山、宝鼎山、十里坪坦等风电场。推进宝工区、大祥、洞口、武冈光伏发电。加快2个220千伏、13个110千伏、9个35千伏、934个10千伏输变电工程建设。坚决完成自供区的未改村农网改造任务。实施“气化邵阳”，启动市区—邵东、市区—邵阳县2条天然气输气管网建设。

进一步完善通讯设施。新建4G基站2000个，实现全市行政村4G信号全覆盖。实施“宽带邵阳”，加快城乡有线和无线宽带建设。清查铁塔、基站机房等存量通信设备的底数，并做好移交工作。

6、全面深化改革，使改革红利转化为加快发展的效益

深化行政事业机构改革。完成市县政府机构改革和事业单位改革。抓紧食药机构职能整合、人财物划转等按时到位。推进公路管理体制改革，将人财物整体下放。落实好省直管县改革试点。稳步推进有条件的乡镇村区划合并调整。

深化经济体制改革。继续深化行政审批制度改革。做好改制企业各类社保接续、社区移交、水电分离改造等工作，年内基本解决市县属国有企业历史遗留问题。全面清理、归集经营性国有资产，基本完成政府出资企业股份制改造。完善国有控股企业监事制度。推进预算编制改革，构建全口径的财政预算体系。完善政府性债务规模、类别“双控”和风险预警机制，有效防范和化解债务风险。稳步推进“营改增”试点范围和资源税改革。大胆试行政府购买服务，鼓励社会资本以特许经营、租赁经营、股权投资等方式参与重点领域建设。深化商事制度改革，全面推行“先照后证”。深化国有林场改革。

深化社会体制改革。健全市级公共文化服务体系建设协调机制。加快经营性文化事业单位转企改制，深化国有文艺院团运行机制改革。探索文化艺术中心管理的新模式、新机制。推进医疗体制改革，综合改革县级公立医院。推进机关事业单位与城镇职工统一的养老保险和工资制度改革。稳妥实施公车改革。

7、切实保障和改善民生

以创新创业带动就业。大力实施“创业引领计划”、“创业扶持计划”，重点扶持高校毕业生特别是邵籍大学生、城镇困难人员和农村富余劳动力就业创业。全年新增城镇就业4.5万人，失业人员再就业2.3万人，完成各类就业培训4.5万人，转移农村富余劳动力稳定在120万人以上。

逐步提升社保层次和水平。以社会保险全覆盖为目标，积极开展全民参保登记工作，实施“五险”统一征缴。认真落实养老保险缴费费率过渡试点政策，进一步完善城乡居民养老保险缴费激励机制。改扩建敬老院8所。建成市第二福利院老年养护楼和残疾人康复中心。

着力改善困难群众住房条件。继续推进棚户区改造五年行动计划，加快资江南岸、龙须塘、小江湖半岛等“十四大片区”改造步伐。建成公租房23177套139万平方米，改造城市棚户区26393户147万平方米、国有工矿棚户区2550户14万平方米。加快农村教职工、医务人员周转房建设。坚决完成省里下达的农村危房改造任务。抓好安置房建设，市区年内建成安置小区11个，搬迁入住6800户以上。争取归集住房公积金17亿元，发放个人住房贷款13亿元。

整体推进扶贫攻坚。实施精准扶贫消除贫困五年行动计划，争取贫困人口人均纯收入增幅高于全市平均水平，减少贫困人口20万人以上。实施1273个贫困村建整扶贫一定三年全覆盖，确保水、电、路、气、房和环境改善“六到农家”。抓好教育扶贫，不让贫困家庭的孩子输在起跑线上，尽力阻断贫困代际传递。

8、统筹推进各项社会事业

提升教育发展水平。扎实推进化解城区大班额三年行动计划，改扩建市区学校17所。建设70所义务教育合格学校和28所农村公办幼儿园。整合职教资源，推进校企合作和中高职融通。鼓励民营资本投资办学。基本实现乡镇及以上中小学校教育信息化“三通二平台”全覆盖。完成邵阳幼专、市一中征地拆迁等前期工作并动工建设。确保邵阳学院与邵阳医专成功合并更名。

强化卫生计生服务。确保市中心医院东院门诊楼主体完工。开工市二人民医院北迁项目和中医院肿瘤综合楼。提升基层医疗机构服务能力，形成基层首诊、分级诊疗、双向转诊的就医新秩序。加强重大传染病防控，确保各项防控措施落实到位。严格控制政策外生育特别是多孩生育，降低出生人口性别比。继续实施优生优育，尽最大努力降低残疾儿出生率。

推进文体事业繁荣发展。采取财政安排、市场运作等多种途径，稳步增加对文化事业的投入。加强文化设施建设，建成文化艺术中心，加快文化旅游产业园等项目建设。加快实施电视报纸“村村通”、广播“村村响”、无线覆盖提质、农村公益电影放映、“送戏下乡”等惠民工程。注重传承和弘扬优秀传统文化，培育和践行社会主义核心价值观。加强国有文物保护。积极参加2015湖南艺术节，筹办好市第三届艺术节。继续支持绥宁侗寨申遗。推进机关和企事业单位使用正版软件。加快市体育中心建设，确保各场馆主体工程全面开工，争取主体育馆竣工。办好第三届市运会，做好承办第十三届省运会的筹备工作。

9、努力形成绿色低碳发展新方式

建设绿色邵阳。启动新一轮“三边三区三年”绿色行动，实施封山育林绿色建设五年行动计划，森林覆盖率稳定在60.6%以上。抓好高铁、高速公路沿线等裸露山体的复绿。抓好矿山生态修复治理和地质找矿工作。实施绿色示范乡村工程，每个县市创建3—5个省级生态乡镇或省级生态村。

有效保护环境。深入实施“十大环保工程”，认真抓好龙须塘等重点区域环境整治。全面推进资江、邵水等重点流域水环境治理，规范采砂洗砂和企业排污行为。严格水资源管理，启动河道保洁工作。加强大气污染防治，重点治理汽车尾气、粉尘等，启动PM2.5监测，加快“煤改气”工程进度。

大力开展节能减排。推进能源资源节约和循环利用，抓好工业、交通、建筑、公共机构等领域节能。淘汰一批落后产能和落后生产工艺，关闭一批高污染、高耗能企业，严格控制主要污染物排放。

10、切实维护社会安全稳定

确保安全发展。牢固树立安全生产红线意识，坚决贯彻落实新修改的《安全生产法》，强化企业主体责任，落实部门监管责任，加强县乡属地管理责任，使党政同责、一岗双责、齐抓共管制度化、常态化。以最严格的监管、最严厉的处罚、最严肃的问责，坚决遏制重特大事故，努力防止和减少一般事故。

推进多层次多领域依法治理。深入推进重大决策社会稳定风险评估工作，着力预防各类重大矛盾冲突。坚决落实五部门文件不打折扣，继续坚持严打整治。加强人防物防技防建设，实现全市电子监控“一张网”。继续推进市（村）民法制学校建设。深入开展依法逐级走访工作，促进信访问题在基层及时就地解决。依法打击信访中的违法犯罪行为，切实维护广大群众合法权益和正常信访秩序，争取赴京非访量下降50%。推动民族宗教事务治理法治化。

加强基层基础工作。按照小事不出村（社区）、大事不出乡镇（街道）的要求，重点抓好乡镇（街道）、村（社区）的安全、治安、信访和交通四支队伍建设，推行工作重心下移，配齐配强人员，确保经费、装备到位，夯实基层基础。推进美好社区建设，做好社区综合服务平台验收工作，发挥村级矛盾纠纷调解委员会和乡规民约的作用。做好第九次社区居委会换届选举。

推进军民融合深度发展。继续加强国防动员、国防教育和民兵预备役建设，做好征兵和双拥工作，争创全国双拥模范城。强化国家安全观念，加大人民防空建设力度，巩固军政军民团结的大好局面，努力为驻邵部队、武警和消防部队创造良好条件。认真落实军转安置任务。

三、以法律和制度规范政府行为

1、强化行政权力的法律约束和制度约束。坚持依法行政，做到“七个坚决”：依法依程序制定更多规则、规定和办法，公布权力清单、责任清单和负面清单，坚决把权力关进制度的笼子里。坚决防止领导签字、表态代替法律、规则、规定和政策。坚决杜绝以言代法、以权压法。坚持重大事项集体决策、科学决策、依法决策，坚决防止个人、少数人说了算。组建统一规范的公共资源交易平台，让公共资源交易阳光规范运作，坚决不搞暗箱操作。进一步规范和加强项目管理，探索建设管理新模式，提高项目管理效率，降低项目建设成本，坚决改变项目预算不准而造成投资随意更改的状况。严格规范行政裁量权基准，坚决防止裁量权过大。

2、自觉接受监督和扩大民主协商。始终坚持党委领导，自觉接受人大及其常委会的法律监督、工作监督和政协的民主监督，充分听取人大代表、政协委员以及各民主党派、工商联、无党派人士、人民团体的意见建议。认真办理人大代表批评建议和政协提案，做到件件有答复、事事有结果。主动接受社会监督和舆论监督，将政府工作置于全社会全程监督之下。深入开展政治协商、行政协商、民主协商、社会协商、基层协商等多种协商活动，建立健全提案、会议、咨询、网络等多种协商方式，切实找到全社会意愿和要求的最大公约数。

3、切实规范和改进行政审批。继续深化行政审批制度改革，推行“一口受理”，不让企业和群众弄不懂、跑断腿、伤透心。实行“限时办理”，真正让行政审批提速提效。严格“规范办理”，避免随意裁量。坚持“透明办理”，给群众一个“明白”。推进“网上办理”，切实方便群众。

4、进一步改进工作作风。牢记作风建设永远在路上，继续聚焦“四风”问题，努力践行“三严三实”。进一步加强公务员队伍建设，着力提高依法行政、开拓创新和服务群众的能力。扎实开展整治“吃空饷”、“庸懒散”和“打牌赌博”三大专项行动，坚决查处一批违反作风建设的人和事。

5、不断加强廉政建设。认真落实党风廉政建设责任制“一岗双责”和廉政准则。加强财政资金监管，公开财政专项资金的用途、审批和执行情况，实现审计全覆盖。严肃查处各类违纪违法行为，始终保持惩治腐败的高压态势。

各位代表，新常态带来新挑战，新挑战孕育新机遇，新机遇开启新征程。让我们紧密团结在以习近平同志为核心的党中央周围，在中共湖南省委和邵阳市委的坚强领导下，凝聚全市人民的智慧和力量，同心同德，锐意进取，真抓实干，为全面建成小康社会和“八个建成”而努力奋斗！
